# Person authentication based on eye-closed and visual stimulation using EEG signals

**DOI:** 10.1186/s40708-021-00142-4

**Published:** 2021-10-11

**Authors:** Hui Yen Yap, Yun-Huoy Choo, Zeratul Izzah Mohd Yusoh, Wee How Khoh

**Affiliations:** 1 Faculty of Information, Science & Technology, Multimedia University (MMU), Melaka, Malaysia; 2 Faculty of Information & Communication Technology, Universiti Teknikal Malaysia Melaka (UTeM), Melaka, Malaysia

**Keywords:** Authentication, Brainwaves, Biometrics, Acquisition protocols, Electroencephalography, ERP

## Abstract

The study of Electroencephalogram (EEG)-based biometric has gained the attention of researchers due to the neurons’ unique electrical activity representation of an individual. However, the practical application of EEG-based biometrics is not currently widespread and there are some challenges to its implementation. Nowadays, the evaluation of a biometric system is user driven. Usability is one of the concerning issues that determine the success of the system. The basic elements of the usability of a biometric system are effectiveness, efficiency and user satisfaction. Apart from the mandatory consideration of the biometric system’s performance, users also need an easy-to-use and easy-to-learn authentication system. Thus, to satisfy these user requirements, this paper proposes a reasonable acquisition period and employs a consumer-grade EEG device to authenticate an individual to identify the performances of two acquisition protocols: eyes-closed (EC) and visual stimulation. A self-collected database of eight subjects was utilized in the analysis. The recording process was divided into two sessions, which were the morning and afternoon sessions. In each session, the subject was requested to perform two different tasks: EC and visual stimulation. The pairwise correlation of the preprocessed EEG signals of each electrode channel was determined and a feature vector was formed. Support vector machine (SVM) was then used for classification purposes. In the performance analysis, promising results were obtained, where EC protocol achieved an accuracy performance of 83.70–96.42% while visual stimulation protocol attained an accuracy performance of 87.64–99.06%. These results have demonstrated the feasibility and reliability of our acquisition protocols with consumer-grade EEG devices.

## Introduction

The growing interest in brain-computer interface (BCI) has led to an increase in the importance of understanding brain functions. BCI refers to a communication pathway between an external device and the human brain without involving any physical movements, and covers both medical and nonmedical uses [1]. Authentication study is one of the examples of BCI which uses brain signals as a biometric identifier. Authentication is essential in our daily lives, which is performed in almost all human-to-computer interactions to verify a user’s identity through passwords, pin codes, fingerprints, card readers, retina scanners, etc.With the growth of technology, advanced biometric authentication has been developed. Physiological biometrics use a person's physical characteristics to identify an individual, such as face, fingerprint, palm print, retina, iris, etc. This type of biometrics is hardly to be replaced once it has been compromised. On the other hand, behavioral biometrics analyze the digital patterns in performing a specific task in the authentication. It is hard to mimic compared with the former biometrics, and it is revocable and replaceable when compromised [2]. While these traditional types of biometrics, human cognitive characteristics can be used to develop an alternative way of conventional physiological and behavioral biometrics [3]. It analyzes an individual’s cognitive behavior (biosignals), such as a person’s emotional and cognitive state for the purpose of identification and verification. 

The motivation of choosing brain signals for authentication lies in the desire for a more privacy-compliant solution compared to other biometric traits. Brain signals possess specific characteristics which are not present in most of the widely used biometrics. They are unique and difficult to be captured by an imposter from a distance, thus increasing their resistance against spoofing attacks. One of the commonly used methods in recording brain signals is Electroencephalography or also known as EEG. It records the brain’s electrical activities by calculating voltage variations within the brain [1]. It is also a straightforward and non-invasive method to record brain electrical activity as it only requires placing electrodes on the scalp’s surface.

Brain activity can be obtained through EEG recordings using specifically designed protocols, including the resting state, motor imaginary, non-motor imaginary and stimulation protocol [4]. The resting-state protocol is easy to operate as it only requires users to rest for a few minutes in either eyes-closed (EC) or eyes-open (EO) state, while the EEG data are recorded. On the other hand, motor imaginary requires the users to mentally simulate a physical action, such as movements of the right hand, left hand, foot and others. Other than that, EEG data can also be acquired by asking the user to perform non-motor imaginary tasks, for instance, mental calculation, internal speech or singing. Finally, the stimulation protocol presents the users with a series of stimuli and the electrical response of the users is recorded. Various stimuli have been proposed and applied in the literature for this protocol, such as pictures, wording, audio, etc.

Despite promising results being reported in the literature, the utilization of EEG-based biometrics system is not currently widespread in practical applications. One of the reasons lies in the implementation and operation of this biometric approach. The performance relies on the design of the acquisition protocol [5]. This approach requires a long period of time for the users to undergo EEG brain’s data recording. This approach is impractical to be used in real life as users would not be willing to spend that much time on the authentication process. Moreover, Ruiz Blondet et al. and Wu et al. [6, 7] argued that most studies used high-density EEG devices, which were very costly and the setup process was time-consuming.

Typically, a biometrics system is expected to be accessed by users frequently. Its fundamental usability elements are effectiveness, efficiency, and user satisfaction [8]. Effectiveness refers to how well a user can perform a task. Efficiency measures how quickly a user can perform the task with a reasonably low error rate. Finally, satisfaction measures the users’ perceptions and feelings towards the application. With these requirements, users may not only need a reliable system, but also a user-friendly and affordable EEG device during the acquisition process. A consumer-grade wireless EEG device with lesser channels can be a potential alternative to replace the clinical-grade device. It should also strike a balance between security and user-friendliness in real-life applications [9]. Thus, the paper aims to propose an acquisition protocol that employs a consumer-grade EEG device with a reasonable enrolment period. In addition, the reliability of the EEG signals recorded via a consumer-grade device is also examined through different sessions with regard to two acquisition protocols, namely eye-closed (EC) and visual stimulation protocol.

The rest of the paper is organized as follows. Section 2 discusses the literature review. Section 3 presents the proposed approach. Section 4 shows the experiment results and performance evaluation, and Sect. 5 discuss es the findings of the proposed system. Finally, Sect. 6 provides a conclusive remark to this paper and some future works are suggested. 

## Related work 

From the beginning of the twentieth century, EEG analysis has been mainly employed in the medication field to study brain diseases such as stroke, brain tumor, epilepsy, Alzheimer, Parkinson, etc. [10]. In particular, it has been heavily employed in BCI in the last decade, where the main objective is to help patients with severe neuromuscular disorders. Applications of BCI functions by either observing the users’ state or allowing the users express their intentions; meanwhile, the users' brain signals are recorded and sent to a computer system for further analysis. The result is then translated into a command and the system is instructed to complete the intended task [1]. Recently, the research of BCI has been extended further to cover several applications, including authentication and security [4].

Cognitive biometric is a new technology that utilizes brain activity to authenticate an individual. The brain’s activity can be recorded by measuring the blood flow in the brain or by measuring the electrical activity of the brain’s neurons. EEG is widely considered for usage in security areas as the signals are unique and possess distinctive characteristics, which are not present in other commonly used biometrics such as face, iris, palm prints and fingerprints. Due to its high privacy compliance nature, EEG-based biometric is robust against spoofing attacks as it is impossible for an imposter to capture the brain signal from a distance [10]. EEG signals are also sensitive to stress. Thus, it is hard to force a person to reproduce brain activity when they are panicked.

In general, biometrics must fulfill four requirements: universality, permanence, uniqueness and collectability [11]. Universality refers to the requirement that each person should naturally possess the characteristic being measured. Permanence requires that the characteristics of a person should stay the same over time for the purpose of criteria matching. Uniqueness is the requirement that the characteristics of a person should be unique and distinguishable from one another. Finally, collectability requires that the characteristics of a person should be measurable with any capturing device. Previous studies had made a significant effort to prove the viability of EEG as a biometric identifier [10, 12–14]. Ruiz Blondet et al. [6] further emphasized that, in terms of collectability, the design of EEG acquisition protocol should be user-friendly to the users. It can be done by reducing the number of electrodes to make the design more feasible and closer to real-world applications. Several EEG acquisition protocols were designed and proposed in the literature to obtain specific brain responses of interest. The main objective was to study the neural mechanisms of information processing in environmental perception and during complex cognitive operations [15]. These acquisition protocols generally be divided into two categories: resting state and stimulation [16].

For the resting state protocol, the user is required to sit on a chair and rest for a few minutes in either eyes-closed (EC) or eyes-open (EO) state as instructed. Meanwhile, the brain signals of the users are recorded. To the best of our knowledge, [17] was the first research that proposed an EEG-based biometric using a resting state protocol. The authors recorded EEG signals from four subjects when they were performing EC activity that lasted for 3 continuous minutes. The spectral values of the signals were calculated using Fast Fourier Transform (FFT). The Alpha frequency band (7–12 Hz) was obtained and this value was further sub-divided into three overlapping sub-bands. The obtained classification scores ranging from 80 to 95% were correct, which proved that the EEG signals can be used as one of the biometric traits. Both sub-bands were informative and no frequency band was reported to have an extra benefit over the others. In La Rocca et al. [10], the repeatability of the EEG signal was addressed. A ‘resting state’ protocol with both EC and EO was designed to acquire raw EEG signals from nine healthy subjects in two different sessions, in which both sessions were 1 to 3 weeks apart. The signals from the 54 electrodes that were attached to the scalp of the subject were continuously recorded. The raw EEG signals were filtered by an anti-aliasing FIR filter before they were presented in four sub-bands from 0.5 to 30 Hz. A common average referencing (CAR) filter was then employed to minimize the artifacts. Each preprocessing signal was modelled according to an autoregressive model while using reflection coefficients to generate the feature vector, then a linear classifier was employed for classification. In the evaluation, a different set of electrodes combination was tested and the results showed a high degree of repeatability over the time interval. In Ma et al. [18], the EEG data were adopted from a public data set. A total of 10 subjects were enrolled and they were asked to perform 55 s of EC and EO tasks, respectively, using a device with 64 electrodes. The recorded EEG signals were segmented into 55 trials separately with a 1-s frame length. 50 trials were used for training and the rest were used for testing purposes. Convolutional neural networks (CNN) was applied for feature extraction and classification. The findings showed that the suggested approach yielded a high degree of accuracy with accuracy of 88% for a 10-class classification. Besides, an inter-personal difference can be discovered using a very low-frequency band of 0 to 2 Hz.

The second EEG acquisition protocol is based on the stimulus of an external event on the subjects. After stimulation, the electrical response of the subjects is recorded through the nervous system. A typically employed stimulation protocol in EEG-based biometric is the Event-Related Potential (ERP). It is a time-locked deflection on the ongoing brain activity after being exposed to an external event. The event can be sensory, visual or audio stimuli [1]. In Palaniappan and Ravi [19], the study was conducted to assess the feasibility of ERP using visual stimuli. 20 subjects participated in the study. Their signals were obtained from 61 electrodes placed on the scalp when they looked at typical black images with white lines of drawn objects such as an aeroplane, a banana, a ball, etc. The recorded signals with an eye blink artifact with magnitude above 100 µV were removed. Besides, those signals were also de-noised through Principal Component Analysis (PCA). The spectra features consisting of power in the gamma band (30 to 50 Hz) were extracted and classified through a Simplified Fuzzy ARTMAP (SFA) neural network (NN). The results showed an average classification of 94.18%, which proved the proposed method’s potential in recognizing individuals.

The stability of the EEG signals was evaluated in [14] using visual stimulation protocol to record raw EEG signals from 45 subjects. Those subjects were presented with several acronyms (example: DVD, TV and TN) which were intermixed with other lexical types. The experiments consisted of three different sessions, which were carried out in 6 months. For the third session, only nine subjects returned for data acquisition. A hardware filter was applied to reduce the influence of DC shifts and bootstrapping was used to generate extra features. Different classifiers such as cross-correlation, support vector machine (SVM) and divergent autoencoder (DIVA) were adopted. The findings verified the permanence of the EEG characteristics and it was found that the brain signals of the subjects could remain stable over a relatively long period of time. Besides, Ruiz-Blondet et al. [20] had suggested that using ERP may provide more accurate results in EEG-based biometric as its elicitation process allows for some control over the user’s cognitive state during EEG data recording sessions. The EEG data from 50 subjects were acquired using 30 sensors. The cognitive Event-Related Potential Biometric Recognition (CEREBRE) protocol was designed to obtain the unique response of the subjects from the brain systems. This protocol includes different categories of stimuli such as sine gratings, low-frequency words, food images, words, celebrities and oddballs. Besides, subjects were also asked to remain in resting state and undergo pass-thought sessions. The duration of the entire experiment was roughly one and a half hours. The study did not apply any artifact rejection or feature extraction method, where only simple cross-correlation was used for classification. The results showed that all stimulus types achieved greater accuracy. In a recent study, the authors in Sabeti et al. [21] investigated the subjects’ features using resting (EO) and ERP acquisition protocol. Each subject was required to perform a task in EO state for 2 min, where no stimulus was imposed for the first task. However, for the second task, audio stimuli were randomly applied and the subjects were requested to discriminate the different pitch levels. The EEG recording for the second task took around 20 min. The EEG signals were filtered using a bandpass filter ranged from 0.5 to 45 Hz. Several features such as spectral coherence, wavelet coefficients and correlation were extracted and evaluated using SVM, K-Nearest Neighbors (KNN) and Random Forest classifiers. Results showed that correlation was the most discriminative feature among other methods in user authentication.

The implementation of the resting protocol from previous studies has shown that the procedure is convenient, but an individual’s mental state is uncontrollable when EEG data are acquired in different sessions. Thus, visual stimulation is proposed to provide more reliable biometric authentication as this approach allows the experimenter to control the individual’s cognitive state during the time of acquisition. However, due to the small size of an ERP, a large number of trials is needed to gain the desired accuracy performance of the authentication, which leads to the users undergoing a lengthy EEG acquisition period [20].

EEG-based systems are still far from being commercialized as they still face several challenges [5]. Usability is one of the challenges which should gain more attention as it is an important principle to determine the success of the system. Users tend to use the system if it is convenient and easy to use. However, most of the current data acquisition process requires a lengthy time to set up, especially for a wired EEG recording device. Besides that, the user has to place a large number of electrodes on their scalp using conductive gel to reduce skin impedance. As an alternative, [7] suggested replacing the cumbersome wired devices with consumer-grade wireless EEG devices which could be more practicable in real life. However, these devices possess a limitation that needs to be considered, where the signal quality could be relatively inferior compared to the research-grade type of devices. Moreover, the lengthy acquisition period is another line of research that needs to be addressed as the participants could lose patience during the acquisition process, which leads to the distortion of the signal or reluctance to take part in the data enrolment process. Therefore, acquisition protocols that utilize a consumer-grade device to acquire EEG signals within a reasonably short period of time are proposed in this work.

## Methods

**Fig. 1:**
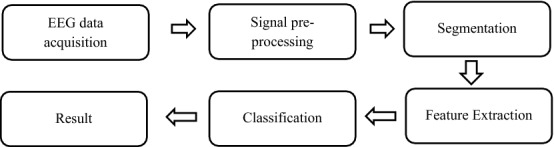
Overflow of proposed method

The performance of an EEG-based biometric depends on a proper design of the acquisition protocol. The portability of the EEG device and acquisition period will be considered to improve the usability and practicability of the system. The proposed system comprises 5 components: data acquisition, preprocessing, signal segmentation, feature extraction, and classification. Figure 1 illustrates the flowchart of the proposed method.

### Acquisition protocol

**Fig. 2:**
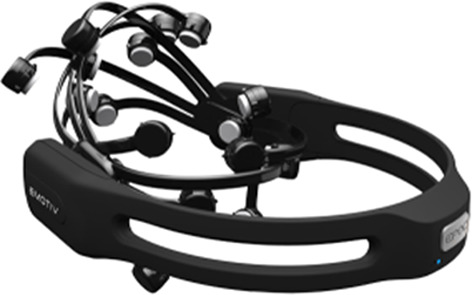
EEG Emotiv EPOC+ wireless headset

**Fig. 3:**
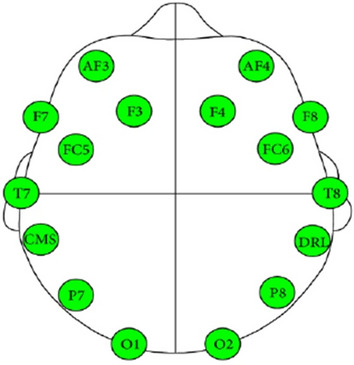
Framework of brainwave user recognition

Conventionally, EEG signals are recorded using clinical-grade EEG equipment. This device is expensive and inconvenient as the setting up could take a tremendous among of time. Hence, in this work, a consumer-grade type of EEG device is used as an alternative to improve user experience. The EEG signals are collected from 8 healthy volunteers (2 female, 6 male, all ages from 18 to 33) using Emotiv EPOC+ wireless headset, as illustrated in Fig. 2. It comprises 14 integrated electrodes with two reference sensors where each sensor is located at the standard positions of the International 10–20 systems as shown in Fig. 3.

Before the acquisition process, a brief introduction about the purpose of the study was given to the subject. In addition, the subject was also allowed to see the changes in their EEG signal when they blinked their eyes or moved their bodies. The purpose of this demonstration is to tell the subject that any eye movements and muscle tension can impact their brain waves. Thus, they were requested to avoid big movements and remain as still as possible. The entire data acquisition process was conducted in a standard enclosed room. The recording process was divided into morning and afternoon sessions to assess the stability of the consumer-grade EEG equipment when recording EEG signals over different sessions. In each session, the subject was required to perform two different tasks (eyes-closed and visual stimulation), while data were recorded at a 256 Hz sampling rate.

**Fig. 4:**
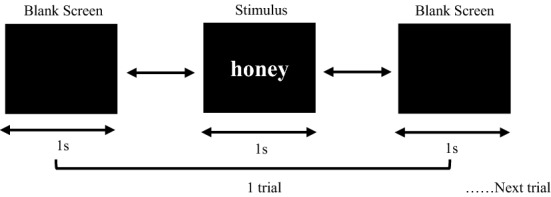
Visual stimulation with using wording presentation

- Task 1: Eyes-closed (EC)—subject was seated on a chair with both arms resting. Before the enrollment, the subject was instructed to keep the mind as calm as possible and remain in a resting state with eyes closed. The recording started 10 s after the subject closed the eyes and remained resting. EEG signals were recorded for 30 s continuously and then the recording process was stopped.
- Task 2: Visual stimulation—the subject was requested to be seated on the same chair without any major movements after completing Task 1. A LED screen of size 17″ was placed in front of the subject. The subject was guided to sit comfortably at a certain distance from the screen. During the recording process, a series of stimuli with 120 single words were displayed to the subject. The subject was requested to focus and interpret each stimulus silently at all times, where no big body movements were allowed. However, they were allowed to blink their eyes to reduce the tiredness during the enrolment process. The stimulation design was mainly focused on wording presentation as the subject’s semantic memory might provide distinctive biometric properties. Each stimulus was a wording that consisted of four to seven letters that the subject could easily understand. A stimulus was displayed on the computer screen for 1 s followed by a 1-s black screen, as illustrated in Fig. 4. It took approximately 4 min to show all the 120 wordings to the user (including the black screen), then the recording process was stopped. Along the process, an Inter-Stimulus Interval (ISI) could be segmented into parts (coined as a trial in this work) that consisted of 0.5 s of black screen, followed by 1 s of stimulus displayed and another 0.5 s of black screen, as illustrated in Fig. 4.

A total of eight subjects contributed to the EEG data acquisition process and a total of four well-collected data sets were obtained from the two sessions as follows:

- Session 1: Eyes-closed data set, *S*1_ec_
- Session 1: Visual stimulation data set, *S*1_s_
- Session 2: Eyes-closed data set, *S*2_ec_
- Session 2: Visual stimulation data set, *S*2_s_

### Preprocessing and segmentation

EEGLAB is an interactive MATLAB toolbox and was implemented in this study for preprocessing and segmentation purposes. Before performing feature extraction, unwanted artifacts and unnecessary information will be removed from the collected EEG signals, therefore improving the signal-to-noise ratio. Filtering is a process to filter continuous EEG data before epoching or artifact removal. Finite Impulse Response (FIR), a linear filter, was adopted to remove the direct current shifts of the recorded EEG signals where the range was set from 1 to 55 Hz. An Automatic Artifact Removal (AAR) was then applied to data set *S*1_s_ and *S*2_s_ to remove the ocular artifacts in the recorded EEG signals. The AAR is one of the toolboxes available in the EEGLAB plug-in [22] and is used to correct the ocular effects within EEG signals. No artifact rejection was applied to *S*1_ec_ and *S*2_ec_ data sets as the EEG signals collected for these data sets were for resting state without eyes and muscle movements.

After the removal of the artifacts, the EEG signals were segmented into small parts, which were named trials. For eye-closed data sets (*S*1_ec_ and *S*2_ec_), the first 5 s of the signal, which contained inconsistency, were discarded. The remaining EEG signals were then segmented into 25 trials, with each trial containing a 1-s frame length (256 sample points). The frame length had been experimentally selected based on the existing study [10]. On the other hand, for visual stimulation data sets (*S*1_s_ and *S*2_s_), the signals were epoched and ERPs were formed for each stimulus starting from − 1000 ms to stimulus onset and lasting for 1000 ms after probe onset (refer to Fig. 4), resulting in 512 sample points for each trial. In other words, each trial contained a 1-s stimulus and it was embedded with 0.5 s of black screen at both the beginning and the end of the trial. After this, epoch rejection was applied to remove some trials that appeared to contain significant artifacts, resulting in a range of 100–120 trials for each subject after the segmentation process.

### Feature extraction

Cross-correlation was considered to process the EEG signals. Cross-correlation is a measure of the degree to which two series are correlated. It measures how closely two different observables are related to each other at the same or different times by considering time lag [23]. If $x\left[ N \right]$ and $y\left[ N \right]$ are two discrete signals where *N* is the length of the signal, then the correlation of $x\left[ n \right]$ with respect to $y\left[ n \right]$ is given as:

1 $$r_{xy} \left[ l \right] = \mathop \sum \limits_{t = - \infty }^{\infty } x\left[ t \right]y\left[ {t - l} \right],$$

where $l$ is the lag or delay which indicates the time-shift and *t* indicates the period of the signal. If both signals are discrete functions of period *N*, then the − ∞ to ∞ can be replaced by an internal of length *N* from *t*_0_ = 0 to *t*_0_ + *N*. The correlation values between the 14 channels of each trial were computed in a pairwise manner. The maximum of the cross-correlation over all trials for each pair was extracted from the correlation values, which was denoted as $\max .$

The variation of the range value corresponding to the features can deteriorate the performance of the overall system. Thus, all features were normalized to the range between 0 and 1, so that each feature contributed proportionately to the final distance. Assuming that a feature is denoted as $x$, the equation for the normalization can be defined as:

2 $$x_{{{\text{norm}}}} = \frac{{x - x_{\min } }}{{x_{\max } - x_{\min } }},$$

where $x_{{{\text{norm}}}}$ is the normalized features. Therefore, a feature vector $v$ is constructed by concatenating all normalized features as:

3 $$v = \left( {\max \left( {x_{{{\text{norm}}}} } \right),\mu \left( {x_{{{\text{norm}}}} } \right),\sigma^{2} \left( {x_{{{\text{norm}}}} } \right)} \right).$$

### SVM classification

A good classification method is essential to accept or reject a claimed person from accessing the system based on an input. An efficient and effective model is necessary for predicting the classes from the data. In general, the learning process is done using the training data chosen from the sample data and their class label. Researchers have widely used SVM to classify EEG signals. SVM is a classification method that involves separating test data with different class labels by learning the structure from the training data and constructing the hyperplanes in a multidimensional space based on that data [24]. SVM adopts a set of mathematical functions that are known as kernels. The function of a kernel is to receive the data as an input and transform it into the desired form. Polynomial SVM is one of the common kernels used in the data classification of non-linear models among the available SVM kernels. It has a good generalization ability and a low learning capacity when the data are non-linearly separated. Since EEG signals are non-stationary and Polynomial SVM had shown a good classification performance in previous EEG studies [25, 26], Polynomial SVM was used in this study as the classifier for classifying EEG patterns.

While this work aims to recognize an individual using EEG signals, it leads to a multi-class SVM prediction. Multiple class prediction is more complex than binary prediction, because the classification algorithm has to consider more separation boundaries or relations [27]. The present study considered two decomposition strategies: (OVO) one-vs-one and (OVA) one-vs-all. OVO is a pairwise classification that maps all data sets that belong to a certain class. It splits a multi-class classification data set into binary classification problems. The number of generated models depends on the number of classes. Consider the formula *n*/(*n* − 1)/2, where *n* is the number of classes. If *n* is equivalent to 5, the total of the generated models is 10. While OVA is also a paired binary class, it splits a multi-class classification data set into one binary classification problem per class. OVA produces the same amount of learned models as the number of classes. If the number of classes is 5, the number of generated models will also be the same [28].

## Experiment results

In the experiment analysis, the *k*-fold cross-validation technique was adopted to generate fair and averaged performance results, where the *k* was set to 5 in this study. Therefore, in this cross-validation, the data were divided into 5 distinct subsets and repeated for 5 iterations. In each iteration, a subset was selected for testing, while the rest of the subsets (*k* − 1) were used for training. It is noted that the distribution of trials for the subset was randomized and the selection of each subset in each iteration for training and testing purposes was mutually exclusive. The average accuracy was determined for each fold. The average accuracy and its standard deviation, which describes the amount of variability or dispersion around the average, are reported in this section.

The experiments were conducted on both morning and afternoon sessions’ data sets. In addition, to assess the stability of the signals across the different sessions, the trials from both sessions were also merged to produce another data set, which was named as the combined sessions, during the evaluation process. The performance metrics including accuracy, precision, sensitivity, specificity and *F*1-score are reported. These metrics were computed based on four parameters: true positive (TP), false positive (FP), true negative (TN) and false negative (FN), where they are derived as follows:

4 $${\text{Accuracy}} = \frac{{{\text{TP}} + {\text{TN}}}}{{{\text{TP}} + {\text{TN}} + {\text{FP}} + {\text{FN}}}},$$

5 $${\text{Precision}} = \frac{{{\text{TP}}}}{{{\text{TP}} + {\text{FP}}}},$$

6 $${\text{Sensitivity}} = \frac{{{\text{TP}}}}{{{\text{TP}} + {\text{FN}}}},$$

7 $${\text{Specificity}} = \frac{{{\text{TN}}}}{{{\text{TN}} + {\text{FP}}}},$$

8 $$F1\;{\text{score}} = \frac{{{\text{precision}}*{\text{sensitivity}}}}{{{\text{precision}} + {\text{sensitivity}}}}.$$

The averaged classification results of 4 data sets with different sessions are summarized in Tables 1, 2 and 3. As observed from Tables 1, 2 and 3, the visual stimulation task outperformed the EC task in three experiments, including the morning, afternoon and combined sessions. It achieved a very promising accuracy performance especially for morning and afternoon sessions (*S*1_S,OVO_ = 96.91%, *S*1_S,OVA_ = 99.06%, *S*2_S,OVO_ = 97.71%, *S*2_S,OVA_ = 99.05%) as compared to the EC task (*S*1_EC,OVO_ = 83.70%, *S*1_EC,OVA_ = 82.73%, *S*2_EC,OVO_ = 86.69%, *S*2_EC,OVA_ = 96.42%). The accuracy performances of visual stimulation for the combined sessions also outperformed the EC task with accuracies of 87.64% (*S*1 + *S*2_S, OVO_) and 96.56% (*S*1 + *S*2_S, OVA_) while the EC task had accuracies of 86.61% (*S*1 + *S*2_EC, OVO_) and 96.41% (*S*1 + *S*2_EC, OVA_) for both OVO and OVA, respectively.

**Table 1:** Experimental results for Task 1 and Task 2 in the morning session, *S*1

Task	Classification measurement (averaged% ± standard deviation)									
	OVO					OVA				
	Acc.	Pre.	Sens.	Spec.	*F*1	Acc.	Pre.	Sens.	Spec.	*F*1
Eye-closed	83.70 ± 4.3	86.58 ± 5.1	83.67 ± 4.5	97.67 ± 0.61	82.73 ± 5.2	96.23 ± 3.33	97.39 ± 3.1	98.44 ± 2.12	81.00 ± 23.30	97.87 ± 1.85
Visual stimulation	96.91 ± 1.57	97.05 ± 1.53	96.77 ± 1.64	99.56 ± 0.22	96.81 ± 1.62	99.06 ± 0.85	99.58 ± 0.56	99.35 ± 0.86	96.83 ± 4.3	99.46 ± 0.48

ACC: accuracy; Pre: precision; Sens: sensitivity; Spec: specificity; *F*1: *F*1 score

**Table 2:** Experimental results for Task 1 and Task 2 in the afternoon session, *S2*

Task	Classification measurement (averaged % ± standard deviation)									
	OVO					OVA				
	Acc.	Pre.	Sens.	Spec.	*F*1	Acc.	Pre.	Sens.	Spec.	*F*1
Eye-closed	86.69 ± 6.20	88.85 ± 5.29	86.67 ± 6.19	98.10 ± 0.89	86.44 ± 6.40	96.42 ± 3.01	97.54 ± 2.43	98.44 ± 2.11	82.17 ± 18.00	97.97 ± 1.70
Visual stimulation	97.71 ± 1.64	97.88 ± 1.36	97.75 ± 1.43	99.67 ± 0.20	97.74 ± 1.44	99.05 ± 0.85	99.58 ± 0.55	99.34 ± 0.85	96.83 ± 4..35	99.46 ± 0.48

**Table 3:** Experimental results for Task 1 and Task 2 in combined sessions, *S*1 + *S*2

Task	Classification measurement (averaged% ± standard deviation)									
	OVO					OVA				
	Acc.	Pre.	Sens.	Spec.	*F*1	Acc.	Pre.	Sens.	Spec.	*F*1
Eye-closed	86.61 ± 2.69	87.21 ± 2.99	86.74 ± 2.64	98.08 ± 0.38	86.75 ± 2.66	96.41 ± 2.62	97.26 ± 2.43	98.72 ± 0.99	80.53 ± 17.53	97.97 ± 1.46
Visual stimulation	87.64 ± 1.66	88.06 ± 1.65	87.76 ± 1.60	98.23 ± 0.24	87.62 ± 1.67	96.56 ± 2.61	97.42 ± 2.41	98.73 ± 0.90	81.68 ± 17.37	98.06 ± 1.46

Based on the comparison, it is noticed that the visual stimulation task performed better than the EC task. Some statistical tests were carried out in this study to measure the significant difference between each task. First, the Shapiro–Wilk Test was used to evaluate the acquisition methods’ results and examine if they obeyed normal distribution. If the data is normally distributed, the paired *t* test is applied to assess the consistency of classification performance; otherwise, the Wilcoxon Rank Sum test is considered. These calculations were performed using the SPSS software. The Shapiro–Wilk test showed that the average classification accuracy for OVO–SVM only obeyed the normal distribution in the morning, afternoon and combined sessions. The probabilities are summarized in Table 4. As the data were a normal distribution, a paired *t* test was conducted to compare the differences of OVO classification measurement between the EC task and the visual stimulation task. The paired *t* test showed that visual stimulation performed better than EC in the morning and afternoon sessions, where both classification measurements (*p* < 0.05) were significant.

**Table 4:** Normal distribution results

Task	Normal distribution probabilities using Shapiro–Wilk test					
	OVO			OVA		
	Morning	Afternoon	Combined sessions	Morning	Afternoon	Combined sessions
Eye-closed	0.871	0.146	0.271	0.001	0.000	0.007
Visual stimulation	0.536	0.297	0.961	0.000	0.001	0.007

On the other hand, based on Table 4, the distribution for OVA classification accuracy did not resemble a normal distribution. Thus, the Wilcoxon Rank Sum test, the non-parametric alternative to the paired *t* test, was applied. The Wilcoxon Rank Sum test results indicate that the visual stimulation’s performances were better than EC in both morning and afternoon sessions. There was a significant difference at the level of 0.05. Meanwhile, visual stimulation also outperformed EC when the morning session was combined with the afternoon session; however, the difference was not significant. The results are reported in Table 5.

**Table 5:** Paired *t* test and Wilcoxon sRank Sum test for classification measurements (*p* values)

Sessions	Paired *t* test (OVO)					Wilcoxon Rank Sum test (OVA)				
	Acc.	Pre.	Sens.	Spec.	*F*1	Acc.	Pre.	Sens.	Spec.	*F*1
Morning	0.005	0.006	0.005	0.015	0.007	0.000	0.014	0.000	0.000	0.000
Afternoon	0.005	0.016	0.017	0.015	0.017	0.000	0.000	0.000	0.000	0.000
Combined sessions	0.012	0.357	0.361	0.443	0.360	0.201	0.864	0.279	0.230	0.310

The results in Table 5 show the range of *p* values from 0.000 to 0.017 in the morning and afternoon sessions. Therefore, there is sufficient evidence to conclude that the classification accuracy can achieve better performance on average if the EEG data are acquired through visual stimulation task in separate time sessions. The visual stimulation task appears to be effective in terms of better capabilities in recognizing the claimed users. However, it was also observed that the combined sessions did not inherit similar characteristics to both previous sessions where the *p* values were not significant (*p* > 0.05). These results imply that the intra-class data variability does not significantly impact the signal’s stability, thus leading to similar results between visual stimulation and EC.

On the other hand, it was also observed that OVA outperformed OVO in most performance metrics for both EC and visual stimulation tasks in all experiments. The finest accuracy for OVA considering both visual stimulation task and EC task was 99.06%. It had a higher average accuracy of 12.5% (EC) and 4.59% (visual stimulation), respectively, compared to OVO. The comparison results between both decomposition strategies in SVM are reported in Table 6. However, it is noticed that there was degradation in terms of specificity performance in OVA for all experiment tasks. It may be due to the OVA strategy which involves duplication of a single binary classification per class, where the samples from a particular class are assigned as positive while samples from the rest of the classes are assigned as negatives for each iteration. Assuming *n* is the number of classes, the OVA repeats for *n* times, and for each time, a class is defined as a positive class while the rest of the classes (*n* − 1) are denoted as negatives. In this way, there is classifier imbalance as the number of negative samples is significantly larger than positive samples. It increases the chance for the system to rule out the negative samples mistakenly, thus decreasing the true-negative result.

**Table 6:** Classification accuracy of OVO and OVA

Task	OVO			OVA			OVA > OVO (%)		
	Morning	Afternoon	Combined sessions	Morning	Afternoon	Combined sessions	Morning	Afternoon	Combined sessions
Eye-closed	83.7	86.69	86.61	96.23	96.42	96.41	14.97	11.22	11.31
Visual stimulation	96.91	97.71	87.64	99.06	99.05	96.56	2.22	1.37	10.78

## Discussion

**Fig. 5:**
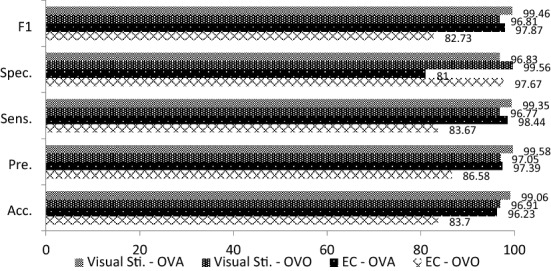
Comparison results of EEG acquisition protocols for the morning session, *S*1

**Fig. 6:**
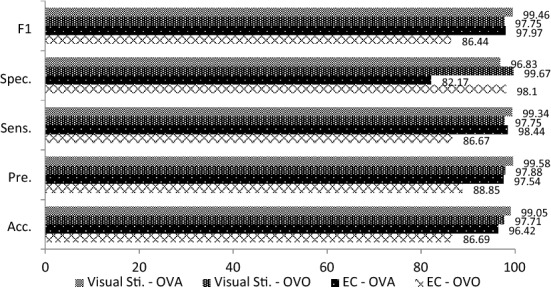
Comparison results of EEG acquisition protocols for the afternoon session, *S*2

**Fig. 7:**
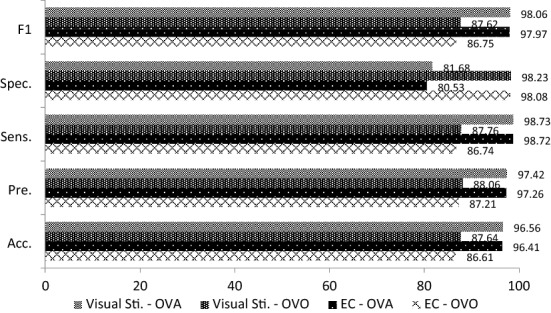
Comparison of EEG acquisition protocols for the morning and afternoon sessions, *S*1 + *S*2

The overall results obtained in this study reveals that EEG signals are an effective biometric identifier in user authentication. As shown in Figs. 5, 6 and 7, the visual stimulation task had better accuracy performance than the EC task. In addition, the results for the EC task had a higher standard deviation than the visual stimulation task. It is believed that the subjects’ minds in EC protocol are uncontrollable without the existence of stimulus, therefore leading to the instability of the signal produced. The findings also reveal that the visual stimulation with ERP protocol is better than EC protocol as ERP allows the experimenter to tightly control the user’s cognitive state. Although performance degradation was observed when combining both morning and afternoon sessions, the specificity was still sustained within 81.68–98.23% and 80.53–98.08% for visual stimulation and EC tasks, respectively. These results indicate that the proposed system is able to identify the ratio of true negatives to total negatives in the data set. A comparison of the proposed method with existing works is listed in Table 7.

**Table 7:** Performance comparison of the existing works

Authors	Acquisition protocol	Type of EEG devices	Wired or wireless	Session	Duration	Performance measure
Ma et al. [18]	Resting-state (EO and EC)	Research/clinical	Wired	1	55 s for each task	Accuracy: 64–88%
Armstrong et al. [14]	ERP (word items)	Research/clinical	Wired	3	Not mentioned	Accuracy: 82–97%
Maria et al. [20]	ERP (images)	Research/clinical	Wired	1	1 and half hour	Accuracy: 100%
Sabeti et al. [21]	− Resting-state with EO − ERP (audio)	Research/clinical	Wired	1	− 2 min − 20 min	Accuracy: EC: 38.20–77.53% ERP: 23.76–99.06%
Proposed method	− Resting-state with EC − ERP (word items)	Consumer	Wireless	2	− 30 s − 4 min	Accuracy: EC: 83.7–96.42% ERP: 87.64–99.06%

As seen from the table above, the proposed method had obtained better results than most existing works. Although the accuracy reported in [20] was perfectly accurate, it is the least practical as the acquisition process took one and a half hours to retrieve EEG responses from individuals based on six types of stimulus. In terms of EEG recording devices, most reported studies preferred using research-grade devices due to their reliability. The proposed method uses a consumer-grade device, which is proven to have the capability to recognize the individuals even in separate sessions. In addition, it is cost-effective in terms of practical applications. Furthermore, the acquisition duration is one of the key reasons that makes the proposed protocol more applicable in a real-world environment. In past works, they took a minimum of 55 s for the EO or EC task, and 20 min for the ERP task. The proposed study reduced the duration to 30 s for the EC task and 4 min for the ERP task, which implies that both cases can achieve very promising results. Moreover, tests for different sessions were conducted to assess the stability of the EEG signals and the results demonstrate the suitability of the proposed acquisition protocol in the authentication field.

## Conclusion

This paper discussed an EEG-based recognition system’s acquisition protocols and performance comparison between the EC and visual stimulation protocols. We proposed using a consumer-grade EEG device for individual authentication in our study. A reasonable acquisition period was proposed to ensure the feasibility of the EEG-based biometric in the future. In this study, cross-correlation was determined to measure the correlation between two different EEG channel signals. We obtained good results when the classification was carried out using cross-correlation together with SVM. The results show that using visual stimulation protocol achieved better performance in terms of classification and consistency than using the EC protocol. However, there is a potential to apply incremental learning to model intra-class variability over time. Besides, OVA performed better than OVO. It can be noted that the distribution for OVA’s classification accuracy did not resemble a normal distribution due to the small size of the samples. Therefore, a non-parametric test was needed to compare the differences of the classification measurement between the proposed methods. The results indicate that visual stimulation performed better than EC in both morning and afternoon sessions with a significant difference at the level of 0.05. Larger sample classes are recommended for further comparison between OVO and OVA. Future works that can be carried out include investigating the extraction and selection of more reliable features from EEG signals with a larger sample size and applying other classification methods to improve the intra- and inter-individual EEG stability.

## Data Availability

Not applicable.

## Abbreviations

AAR: Automatic Artifact Removal
BCI: Brain Computer Interface
CNN: Convolutional neural network
DIVA: Divergent Auto Encoder
EC: Eyes closed
EEG: Electroencephalogram
EO: Eyes Open
ERP: Event-Related Potential
FFT: Fast Fourier Transform
FN: False negative
FP: False positive
KNN: K-Nearest Neighbors
NN: Neural network
OVA: One-vs-all
OVO: One-vs-one
PCA: Principal Component Analysis
SVM: Support vector machine
TN: True negative
TP: True positive
